# Health-Related Quality of Life in Children with Acute Leukaemia and Central Nervous System Tumours: Current Evidence and Future Directions

**DOI:** 10.3390/children13091186

**Published:** 2026-09-03

**Authors:** Lyazat Manzhuova, Timur Saliev, Aigul Bazarbayeva, Maira Kabakova, Gulzhan Kashaganova, Aizhan Kudaibergenova, Zaure Dushimova

**Affiliations:** 1 Scientific Centre of Paediatrics and Paediatric Surgery, Al-Farabi Avenue 146, Almaty 050060, Kazakhstan; 2 Institute for Fundamental and Applied Medical Research, S.D. Asfendiyarov Kazakh National Medical University, Tole Bi Street 94, Almaty 050000, Kazakhstan; 3 Department of General and Applied Psychology, Al-Farabi Kazakh National University, Almaty 050040, Kazakhstan; 4 Department of Cybersecurity, Institute of Automation and Information Technologies, Satbayev University, Almaty 050013, Kazakhstan; 5 Department of Fundamental Medicine, Al-Farabi Kazakh National University, Almaty 050040, Kazakhstan

**Keywords:** health-related quality of life, acute lymphoblastic leukaemia, acute myeloid leukaemia, central nervous system tumours, paediatric oncology, survivorship

## Abstract

**Highlights:**

**What are the main findings?**

- CNS tumour survivors have worse HRQoL than leukaemia survivors; ALL survivors near norms, AML understudied.
- Heterogeneous measurement tools, scarce longitudinal data, and under-representation of key groups limit evidence.

**What are the implications of the main findings?**

- Clinical care should integrate standardised HRQoL assessment, stratified survivorship models, and interventions addressing family/socioeconomic barriers.
- Research must prioritise longitudinal, multisite studies with harmonised measures and inclusive recruitment to ensure equity and meaningful life participation.

**Abstract:**

Remarkable advances in paediatric oncology over recent decades have transformed acute leukaemia and central nervous system (CNS) tumours from largely fatal diagnoses to conditions with survival rates exceeding 80% in many cases. This epidemiological success, however, has shifted the clinical and research focus toward survivorship and the long-term consequences of curative therapies. Health-related quality of life (HRQoL) has emerged as a critical outcome measure that captures the multidimensional impact of disease and treatment on children’s physical, psychological, social, and educational functioning. This review synthesises current evidence on HRQoL in children with acute leukaemias, particularly acute lymphoblastic leukaemia (ALL) and acute myeloid leukaemia (AML), and CNS tumours, the two most common childhood malignancies. We examine measurement approaches, disease-specific HRQoL patterns, comparative outcomes between diagnostic groups, biopsychosocial determinants, and emerging interventions. The evidence consistently demonstrates that while ALL survivors generally experience HRQoL comparable to or slightly below population norms, children with CNS tumours face substantially greater and more persistent impairments across multiple domains. Treatment-related factors, particularly cranial irradiation, neurocognitive sequelae, family functioning, and socioeconomic status emerge as key determinants.

## 1. Introduction

The past five decades have witnessed a dramatic transformation in the outlook for children diagnosed with cancer. Contemporary treatment protocols now achieve cure rates exceeding 80% among children receiving modern therapies [1]. Among the most common paediatric malignancies, acute lymphoblastic leukaemia (ALL) accounts for approximately 25% of all childhood cancers, with survival rates now surpassing 90% [2,3]. Central nervous system (CNS) tumours represent the second most frequent diagnostic category and the leading cause of cancer-related mortality in children [4]. Cure rates for CNS tumours vary considerably by histological subtype, ranging from over 90% for low-grade gliomas to approximately 60–70% for high-risk medulloblastomas and other embryonal tumours [4]. This heterogeneity in survival outcomes, which contrasts with the more uniformly favourable prognosis for ALL, underscores the need for diagnosis-specific considerations when evaluating HRQoL.

This success has, however, introduced a new set of challenges. The very therapies that cure children, intensive chemotherapy, cranial irradiation, and surgery, can inflict lasting damage on developing brains and bodies. Survivors face a broad spectrum of late effects that extend beyond the biological to encompass profound psychological and social dimensions [5]. These include neurocognitive impairments, endocrine dysfunction, secondary malignancies, cardiovascular disease, psychosocial difficulties, and compromised educational and vocational attainment.

Health-related quality of life (HRQoL) has consequently become an indispensable outcome measure in paediatric oncology. Unlike traditional biomedical endpoints such as survival or disease-free intervals, HRQoL captures the patient’s subjective experience of health across physical, emotional, social, and role-functioning domains [6]. For children and adolescents, HRQoL assessment must also account for developmental stage, the perspective of parents and caregivers, and the unique challenges of growing up with a life-threatening illness [7].

Throughout this review, “children undergoing active treatment” refers to patients currently receiving cancer-directed therapy; “early post-treatment period” denotes the first five years after treatment completion; and “long-term survivors” refers to individuals who have survived five or more years since diagnosis. Where the original evidence does not permit such distinction, the term “survivors” is used broadly to encompass all post-diagnosis patients.

This review focuses specifically on HRQoL in children with acute leukaemias and CNS tumours, the two most extensively researched diagnostic groups in paediatric psycho-oncology. We aim to: (1) synthesise current evidence on HRQoL outcomes across these populations; (2) compare HRQoL between diagnostic groups and with healthy peers; (3) identify biopsychosocial and clinical determinants; (4) evaluate existing interventions; and (5) propose future research and clinical directions.

### Literature Search Strategy

This narrative review synthesises evidence on health-related quality of life in children with acute leukaemias and central nervous system tumours. A structured literature search was conducted in PubMed, Scopus, and Web of Science for publications from January 2000 to December 2025. The search strategy combined keywords related to the population (“paediatric”, “child”, “adolescent”), condition (“acute lymphoblastic leukaemia”, “acute myeloid leukaemia”, “central nervous system tumours”, “brain tumours”), and outcome (“health-related quality of life”, “HRQoL”, “quality of life”). Additional articles were identified by manual screening of reference lists of included studies and relevant reviews. As this is a narrative review, no formal quality assessment or meta-analytic synthesis was performed; findings were synthesised thematically across diagnostic groups and outcome domains.

## 2. Measuring HRQoL in Paediatric Oncology

### 2.1. Conceptual Framework

Health-related quality of life is a multidimensional construct encompassing physical health, psychological wellbeing, social relationships, and the capacity to perform age-appropriate functional roles. In children, assessment must account for developmental stage, as cognitive understanding of health and illness evolves from concrete symptom-focused perceptions in early childhood to more abstract reasoning in adolescence [8]. Consequently, the relative salience of each HRQoL domain shifts across developmental stages: physical functioning and freedom from pain may dominate the concerns of younger children, whereas social acceptance, school performance, and emerging autonomy become increasingly prominent for adolescents. Moreover, the impact of disease and its treatment on quality of life is mediated by developmental tasks such as identity formation, educational attainment, and peer belonging, making it essential that both assessment and interpretation are grounded in a life-span developmental perspective that recognises the changing priorities and vulnerabilities of growing children [9].

### 2.2. Assessment Instruments

A diverse array of instruments has been developed to capture HRQoL in children with cancer, reflecting the broad recognition of this outcome as a core endpoint in paediatric oncology research and clinical care. The Paediatric Quality of Life Inventory (PedsQL) 4.0 Generic Core Scales and its accompanying 3.0 Cancer Module represent the most extensively used and psychometrically robust measures, demonstrating strong reliability across diverse populations, with internal consistency reported as Cronbach’s alpha of 0.96 for the total scale and values ranging from 0.82 to 0.95 across individual subscales [10]. Its validation in numerous languages and cultural settings facilitates meaningful cross-national and cross-cultural comparisons, making it a preferred tool in international collaborative studies. Other commonly employed instruments include the Child Health Questionnaire, which provides comprehensive coverage of physical and psychosocial domains; the Health Utilities Index, which is particularly valued for generating preference-based utility scores suitable for cost-utility analyses; and the Pediatric Oncology Quality of Life Scale, which offers a cancer-specific perspective [11]. However, a recent meta-analysis of comparative studies in paediatric haematological malignancies identified no fewer than twelve distinct HRQoL tools across thirty included investigations, highlighting a profound heterogeneity in measurement approaches that currently complicates direct comparability across studies, hampers the synthesis of evidence through meta-analysis, and underscores the urgent need for greater harmonisation and consensus in instrument selection.

### 2.3. Self-Report Versus Proxy-Report

A recurrent and methodologically critical question in paediatric HRQoL research concerns the relative merits of child self-report versus parent-proxy report, each offering a distinct window onto the child’s experience. Substantial evidence now demonstrates that children as young as five years can reliably and validly self-report their HRQoL when developmentally appropriate, age-tailored instruments are used, supporting the inclusion of paediatric perspectives whenever cognitively feasible. Nonetheless, parent–child agreement remains only moderate at best, with parents consistently tending to report more impairment and functional limitation than children themselves, particularly in the psychosocial and emotional domains, a discrepancy that may reflect parental distress, caregiver burden, protective projection, or differences in observational contexts [11]. These perspectives are complementary: self-reports capture the child’s subjective experience, while proxy reports provide an external view across settings. Contemporary guidelines recommend obtaining both informants where feasible, as discordance may signal unmet supportive care needs, and the dual approach enhances clinical validity [11,12].

### 2.4. Methodological Challenges

Despite the conceptual and methodological advances achieved in recent decades, several persistent and emerging challenges continue to complicate HRQoL measurement in paediatric oncology, limiting the robustness and clinical translation of existing evidence. First, the marked heterogeneity in instrument selection, ranging from generic health profiles to cancer-specific modules and utility-based indices, severely limits the comparability of findings across individual studies and compromises the reliability of meta-analytic syntheses, making it difficult to derive definitive, broadly applicable conclusions. Second, there is no established consensus regarding what constitutes a clinically meaningful difference in HRQoL scores within paediatric cancer populations, which hampers the interpretation of change over time and the evaluation of intervention effectiveness in both clinical trials and routine practice [13,14]. Third, the evidentiary base remains heavily weighted toward cross-sectional investigations, while rigorous longitudinal studies that track HRQoL trajectories from diagnosis through active treatment and into long-term survivorship remain relatively scarce, thereby limiting our understanding of critical transition periods and optimal timing for supportive interventions [15]. Fourth, specific and vulnerable subgroups, including infants and very young children who cannot self-report, adolescents facing unique psychosocial transitions and loss of peer connections, and patients from low- and middle-income countries where cultural and linguistic adaptations are often absent, remain consistently under-represented, significantly threatening the generalisability of available data (Table 1).

## 3. HRQoL in Acute Lymphoblastic Leukaemia

### 3.1. HRQoL During Treatment

Children undergoing treatment for acute lymphoblastic leukaemia (ALL) face intensive, multi-modal regimens that include corticosteroids, vincristine, asparaginase, and central nervous system-directed therapy, often administered over two to three years. During active treatment, health-related quality of life (HRQoL) is substantially impaired across all domains, physical, emotional, social, and school functioning. A systematic review of 14 studies representing 1254 paediatric patients found that HRQoL during treatment was significantly lower than both normative data and healthy control groups [23]. The physical domain is particularly affected, with children experiencing fatigue, nausea, pain, weight gain, and reduced mobility due to corticosteroid-induced myopathy and neuropathy from vincristine. Psychosocially, treatment disrupts normal childhood activities, including school attendance, peer interactions, and participation in sports and hobbies. Hospitalisations, frequent clinic visits, and treatment-related side effects contribute to social isolation and emotional distress, not only for the child but also for parents and siblings [24]. Furthermore, methotrexate (MTX)-induced neurotoxicity, manifesting as leukoencephalopathy, seizures, or stroke-like episodes, and posterior reversible encephalopathy syndrome (PRESS), associated with calcineurin inhibitors or high-dose chemotherapy, represent additional neurological complications in ALL patients that can lead to severe central nervous system sequelae and further impair HRQoL during treatment [25].

Importantly, the intensity of treatment phases influences HRQoL differentially; induction therapy, with its high-intensity chemotherapy and associated acute toxicities, is associated with the lowest HRQoL scores, followed by consolidation and maintenance phases, where some recovery is observed. High-dose chemotherapy, especially with marrow ablation in preparation for hematopoietic stem cell transplantation, is also associated with poorer outcomes, reflecting the cumulative toxicity of intensive conditioning regimens that can cause both acute and chronic organ damage, including pulmonary fibrosis, hepatic dysfunction, and endocrine insufficiency [26]. The combination of high-dose chemotherapy and total body irradiation, used in some transplant protocols, produces particularly severe late effects, including secondary malignancies, infertility, and profound neurocognitive impairment.

Recent evidence suggests that integrated psychosocial support and symptom management protocols during active treatment can mitigate some of these HRQoL impairments, though significant deficits remain [23,27]. The substantial burden of treatment underscores the critical need for routine HRQoL monitoring and early supportive care interventions tailored to the specific challenges of each treatment phase.

### 3.2. Early Post-Treatment Period (Up to Five Years)

The early post-treatment phase represents a period of adjustment, recovery, and gradual reintegration into normal life. Completing treatment signalled a significant improvement in HRQoL compared to being on treatment. However, HRQoL remained significantly lower than the general population during this early post-treatment period. Several factors contribute to this persistent HRQoL deficit [28]. Treatment-related late effects begin to emerge, including neurocognitive impairment (particularly in those who received cranial irradiation), endocrine dysfunction (growth hormone deficiency, thyroid dysfunction, and metabolic syndrome), cardiac toxicity from anthracyclines, and secondary bone health issues [29]. Survivors often experience fatigue, which may persist or even worsen after treatment completion, a phenomenon known as “post-treatment fatigue.” Psychologically, the transition from active treatment to survivorship brings unique challenges, including anxiety about disease recurrence, difficulty reintegrating into school and social settings, and adjustment to altered physical appearance or functional limitations [30]. Lower HRQoL in this period is associated with demographic factors (older age at diagnosis and female sex), family dysfunction (parental distress, financial strain, and marital conflict), and treatment-related factors (higher cumulative doses of corticosteroids, cranial irradiation, and anthracyclines) [31]. Notably, family functioning has emerged as a significant predictor of HRQoL outcomes, with cohesive, well-adjusted families being associated with better outcomes and potentially buffering against psychosocial morbidity. This early post-treatment period is therefore a critical window for targeted interventions, including cognitive rehabilitation, psychological support, and transition care programs designed to address the multifaceted needs of survivors and their families.

### 3.3. Long-Term Survivorship

ALL survivors constitute the largest group of childhood cancer survivors, with five-year survival rates now exceeding 90% in high-income countries. A systematic review conducted by Vetsch et al. following PRISMA guidelines identified 31 studies representing 4356 survivors and 901 proxies, providing a comprehensive picture of long-term HRQoL outcomes [32]. The findings reveal a nuanced and somewhat contradictory picture: thirteen studies found worse overall HRQOL scores compared with healthy controls or population norms, eight found no significant difference, and three found better scores, a phenomenon sometimes referred to as “response shift,” whereby survivors recalibrate their expectations and derive greater appreciation for life. Overall, ALL survivors typically had better overall HRQOL scores than survivors of other childhood cancers, particularly those with central nervous system tumours or sarcomas, reflecting differences in treatment intensity and late effect profiles [33].

Importantly, clinical variables such as treatment received were not consistently associated with HRQOL. However, experiencing worse late effects was strongly associated with lower HRQOL. Survivor and parent socio-demographic factors and psychological factors such as resilience and depression were also associated with HRQOL.

### 3.4. Acute Myeloid Leukaemia

Compared to ALL, evidence on HRQoL in paediatric acute myeloid leukaemia (AML) is relatively limited, reflecting both the lower incidence of AML (approximately 20% of childhood leukaemias) and the historically lower survival rates, which have only recently approached 70–80% with intensive chemotherapy regimens [34]. The treatment for AML is typically more intensive and shorter in duration than ALL, involving high-dose cytarabine, anthracyclines, and often hematopoietic stem cell transplantation, a procedure associated with significant acute and chronic morbidity. A five-year prospective study from China (by Wu et al.) demonstrated that early impairment of HRQoL in paediatric AML patients has predictive value for long-term prognosis, suggesting that HRQoL assessment may serve as a valuable prognostic tool [35]. Household income has been examined as a potential determinant, though findings are complicated by possible selection bias, with studies from lower-resource settings suggesting that financial constraints impact treatment adherence, nutrition, and access to supportive care, all of which affect HRQoL.

Getz and colleagues conducted a large, multi-centre cohort study comparing outpatient versus inpatient neutropenia management in children with AML [36]. Their findings challenge the conventional practice of mandatory hospitalisation until neutrophil recovery, demonstrating that outpatient management was not associated with higher bacteremia incidence, treatment delays, or worse health-related quality of life. Notably, 86% of families across both strategies expressed satisfaction, though the drivers of dissatisfaction differed: concerns about hospital-acquired infections and family separation drove dissatisfaction with inpatient care, while the stress of home care drove dissatisfaction with outpatient management. However, the authors caution that course-specific mortality during intensification II was higher in the outpatient group, suggesting that this approach may not be suitable for all treatment phases or patients. The study underscores the need for careful patient selection and standardised protocols, while supporting a shift toward more flexible, family-centred care models.

Pawińska-Wąsikowska and colleagues addressed the question of whether adolescents with AML require a special therapeutic approach compared to younger children [37]. In a retrospective analysis of 220 patients treated according to BFM protocols, they found that overall survival at five years did not differ significantly between adolescents and children aged 1 to 15 years. However, relapse-free survival was shorter in adolescents, and treatment-related mortality tended to be higher, though this difference was not statistically significant. Importantly, high-risk genetics and a leukocyte count above 100,000/μL at diagnosis, rather than age above 15 years, emerged as unfavourable prognostic factors for survival. The authors conclude that therapy intensity should be adjusted to individual genetic risk rather than age alone, and that targeted therapies should be considered when indicated.

### 3.5. Cancer-Related Fatigue Across Diagnostic Groups

Cancer-related fatigue is one of the most common and distressing symptoms experienced by children and adolescents with cancer and represents an important determinant of health-related quality of life (HRQoL). Its severity is dynamic and may vary substantially according to diagnosis, treatment phase, medication exposure, and changes in the clinical condition. Tilga and colleagues examined cancer-related fatigue, one of the most common and distressing symptoms in paediatric oncology, using daily patient-reported outcomes collected via the ePROtect app in a prospective cohort of 104 children and adolescents undergoing chemotherapy [38]. The study included more than 11,600 daily assessments, allowing the researchers to capture changes in fatigue over time and identify diagnosis- and treatment-specific patterns that may be missed by conventional, infrequent assessments. The highest levels of fatigue at diagnosis were observed among patients with non-Hodgkin lymphoma and acute myeloid leukaemia (AML), whereas patients with central nervous system (CNS) tumours reported comparatively lower levels of fatigue. Glucocorticoid exposure was strongly associated with increased fatigue, particularly among children with acute lymphoblastic leukaemia (ALL) during steroid-intensive treatment phases. Fatigue was generally lower during outpatient treatment but increased substantially during unplanned hospital admissions, suggesting that acute clinical deterioration and treatment-related complications may contribute to symptom burden. In contrast, periods of immunotherapy were associated with significant improvement in fatigue.

These findings emphasise that fatigue should not be considered a static symptom but rather a dynamic outcome influenced by diagnosis, treatment intensity, medication exposure, and healthcare utilisation. Continuous or repeated patient-reported monitoring may therefore provide clinically valuable information for identifying periods of increased symptom burden and enabling earlier supportive interventions. Incorporating real-time symptom assessment into routine clinical workflows could facilitate diagnosis-specific fatigue management, including optimisation of symptom control, psychosocial support, and adjustment of supportive care during high-risk treatment periods.

Nevertheless, the available evidence remains limited, particularly for paediatric AML, and the heterogeneity of treatment regimens and patient populations makes comparisons between diagnostic groups difficult. Larger, multicentre prospective studies with longitudinal follow-up are needed to better define fatigue and HRQoL trajectories from diagnosis through active treatment and long-term survivorship. Such studies should also evaluate the contribution of specific treatment exposures, hospitalisations, and psychosocial factors to persistent fatigue and determine whether early identification and targeted management of fatigue can ultimately improve HRQoL and other long-term outcomes.

## 4. HRQoL in Central Nervous System Tumours

### 4.1. The Unique Burden of CNS Tumours

Children with central nervous system (CNS) tumours face a particularly heavy and complex burden that distinguishes them from other paediatric cancer populations. Unlike leukaemia, where the primary pathology is haematological and treatment-related toxicities primarily affect the blood, bone marrow, and systemic organs, CNS tumours directly affect the brain, the organ responsible for cognition, emotion, behaviour, and personality. The tumour itself may cause focal neurological deficits, raised intracranial pressure, seizures, and cognitive impairment before treatment even begins [39]. Treatment often involves neurosurgical resection, which, depending on tumour location, can cause direct damage to eloquent brain regions responsible for motor function, language, memory, or executive function. This is compounded by radiotherapy, which, despite its efficacy, can impair neurodevelopment, particularly in young children whose brains are undergoing rapid myelination and synaptic pruning. The developing brain is exquisitely sensitive to radiation, with younger age at treatment being the strongest predictor of subsequent neurocognitive deficits [40,41]. Furthermore, chemotherapy, while less neurotoxic than radiation, can still contribute to white matter injury and cognitive dysfunction through mechanisms including oxidative stress, inflammation, and disruption of neural progenitor cell proliferation. The cumulative effect of these factors is a constellation of impairments that affect every aspect of a child’s life, academic achievement, social relationships, emotional regulation, and eventual vocational independence. Unlike many other cancers where survivors may achieve near-normal functioning, CNS tumour survivors often face lifelong disability, with profound implications for patients, families, and healthcare systems.

### 4.2. HRQoL Outcomes in CNS Tumour Survivors

Baqai and colleagues conducted a systematic review and meta-analysis focusing specifically on survivors of paediatric medulloblastoma, the most common malignant brain tumour in children [42]. Their analysis of 12 studies revealed that medulloblastoma survivors have among the lowest quality of life scores compared to survivors of other posterior fossa tumours, particularly when compared to low-grade gliomas, though other embryonal tumours may carry similarly poor or worse prognoses and sequelae. A key finding was the significant discrepancy between self-reported and caregiver-reported outcomes, with survivors rating themselves higher across several domains, including physical, psychosocial, and school functioning. Importantly, the meta-analysis found no significant difference in quality of life between those treated with proton versus photon (conventional) radiation, suggesting that factors beyond radiation modality, such as disease severity, metastatic disease, lower family income, and younger age at diagnosis, may be more critical determinants of long-term outcomes.

In another study, Rüther and colleagues addressed the question of whether tumour location within the central nervous system influences health-related quality of life outcomes [43]. Their systematic review of 16 studies encompassing 1391 participants found surprisingly little evidence for a significant difference between supratentorial and infratentorial tumour locations, with only one study reporting a statistically significant association. The authors identified critical limitations in the existing evidence base, including poor statistical reporting, ambiguous construct definitions, and insufficient adjustment for confounding variables. They concluded that recovery from a paediatric brain tumour extends well beyond the post-treatment period, and that further research into the factors influencing survivor quality of life, including the potential role of tumour location, is urgently needed.

Rothmund and colleagues undertook a broader systematic review to update the conceptual model of health-related quality of life in children with cancer [44]. Drawing from 221 studies, 96 patient-reported outcome measures with 2641 items, and 798 quotations from 45 qualitative studies, they found that 94.8% of identified issues could be mapped onto the existing conceptual framework. However, they proposed important additions to the model, including subdomains for ‘treatment burden’, ‘treatment involvement’, and ‘financial issues’. Notably, physical and psychological aspects were more frequently covered than social issues, pointing to a potential gap in current quality of life assessment.

This heterogeneity underscores the need for standardised HRQoL assessment in clinical practice and research, as well as the importance of considering individual patient characteristics when interpreting HRQoL data.

### 4.3. Neurocognitive and Psychosocial Sequelae

Paediatric brain tumour survivors are at significantly increased risk of cognitive, psychosocial, and educational/vocational sequelae that profoundly impact HRQoL. These patients suffer from neurocognitive impairment, treatment-related side effects, and experience compromised quality of life, affecting academic endeavours, social interaction, and mental wellbeing. The neurocognitive profile is characterised by deficits in processing speed, attention, working memory, executive function, and visuospatial skills, domains essential for academic success and everyday functioning [45]. These deficits are often progressive, with some studies demonstrating a “growing into deficit” phenomenon whereby cognitive function fails to develop at the expected rate, resulting in widening discrepancies between survivors and their peers over time.

Recent empirical evidence from diverse populations further supports these findings. A case–control study from Greece by Mavrea and colleagues (2026) demonstrated that childhood cancer survivors exhibited significant impairments in cognitive functions, including attention, processing speed, and executive function, compared to healthy controls [46]. Importantly, the study also found elevated rates of psychosocial difficulties, including anxiety, depression, and social withdrawal, which were closely associated with cognitive deficits. These findings, derived from a Southern European population, complement existing evidence from North American and Northern European cohorts and highlight the cross-cultural consistency of neurocognitive and psychosocial sequelae in childhood cancer survivors.

The systematic review/meta-analysis conducted by Lassaletta et al. included 10 studies involving 630 children and adolescents with brain tumours treated with either proton beam radiotherapy (PBRT) or photon radiotherapy (XRT) [47]. Patients treated with proton therapy demonstrated significantly better performance on several neurocognitive outcomes. However, cognitive abilities were affected irrespective of treatment modality, especially in those receiving early-stage radiotherapy, suggesting that even proton therapy cannot fully eliminate neurocognitive risk [48]. Psychosocial impacts are equally concerning, with survivors demonstrating low self-esteem, depressive symptoms, increased suicidal ideation, and social isolation. Academic outcomes are often poor, with many survivors requiring special educational support, and long-term vocational outcomes are compromised, with lower rates of employment and independent living compared to siblings and population norms. The combined burden of cognitive and psychosocial impairments creates a cycle of disadvantage, whereby cognitive limitations lead to academic difficulties, which in turn contribute to social isolation and mental health problems, further impairing quality of life.

### 4.4. The Role of Tumour Location

The location of the primary tumour within the CNS appears to influence HRQoL outcomes, reflecting the differential functional impact of lesions in various brain regions. A systematic review evaluating supratentorial versus infratentorial tumours found that recovery from a paediatric brain tumour extends beyond recovery post-treatment, and further study into the factors influencing survivor HRQoL, including tumour location, is necessary. Infratentorial tumours, such as cerebellar tumours, are particularly associated with cerebellar mutism syndrome, characterised by transient or permanent speech impairment, ataxia, and emotional lability, which significantly impairs communication and social interaction [49]. In contrast, supratentorial tumours may affect higher-order cognitive functions, with tumours in the frontal lobes producing executive dysfunction, those in the temporal lobes affecting memory and language, and those in the parietal lobes impacting visuospatial skills [50]. These site-specific deficits have downstream effects on HRQoL through their impact on academic performance, employment, and social relationships. Medulloblastoma, the most common malignant brain tumour in children, has been the subject of a systematic review and meta-analysis concluding that quality of life is compromised in all paediatric survivors of medulloblastoma. This highly aggressive tumour typically arises in the cerebellum and is treated with maximal surgical resection, craniospinal irradiation, and chemotherapy [51]. The combination of cerebellar damage and widespread irradiation produces a particularly severe neurocognitive phenotype characterised by deficits in processing speed, attention, and executive function, with consequent impairments in HRQoL across all domains. Understanding the relationship between tumour location and HRQoL outcomes is essential for developing targeted rehabilitation strategies and for counselling families about expected functional outcomes.

### 4.5. Cranial Irradiation and HRQoL

Cranial irradiation is consistently associated with HRQoL deficits in childhood cancer survivors, representing one of the most significant modifiable risk factors for poor long-term outcomes. Research investigating radiation dose to brain substructures has demonstrated that dose-effects, particularly in the pituitary gland and left hippocampus, might contribute to reduced quality of life in survivors of childhood brain tumours. The hippocampus, a structure critical for memory formation and spatial navigation, is exquisitely sensitive to radiation, with doses as low as 10 Gy associated with neurocognitive decline [52]. The pituitary gland, central to endocrine regulation, is sensitive to radiation-induced damage, leading to growth hormone deficiency, hypothyroidism, and other endocrine sequelae that affect physical development and metabolic health. Radiation-induced vascular injury, including microangiopathy and accelerated atherosclerosis, contributes to white matter damage and secondary stroke risk, further impacting cognitive function and HRQoL [53]. These findings highlight the importance of radiation-sparing strategies, including the use of proton beam therapy, which delivers less integral dose to normal tissues, and intensity-modulated radiotherapy, which allows for more precise dose sculpting. Proton therapy has been associated with better neurocognitive outcomes, reduced endocrine dysfunction, and lower rates of secondary malignancies compared to photon therapy, though long-term data remain emerging. Additionally, pharmacological strategies such as neuroprotective agents (e.g., memantine, donepezil) are being investigated for their potential to mitigate radiation-induced cognitive decline. The evidence strongly supports the adoption of radiation-sparing approaches whenever oncologically appropriate and underscores the need for careful radiation planning that considers not only tumour control but also long-term brain health and quality of life. For survivors who have already received cranial irradiation, rehabilitation strategies including cognitive remediation therapy, pharmacological support, and educational accommodations are essential to optimise functional outcomes and HRQoL (Table 2).

## 5. Comparative HRQoL: Leukaemia Versus CNS Tumours

### 5.1. Consistent Differences

A consistent finding across the literature is that children with CNS tumours experience worse HRQoL than those with leukaemia. This difference is not merely statistical but clinically meaningful, with effect sizes that underscore the uniquely devastating impact of brain tumours on paediatric patients and their families [58]. Children with CNS tumours are at increased risk for higher burden compared to leukaemia survivors, largely because of cranial irradiation exposure, which produces a cascade of neurocognitive, endocrine, and psychosocial sequelae that accumulate and often worsen over time. Cranial irradiation, particularly when delivered to young children, disrupts normal brain development, impairing myelination, neurogenesis, and synaptic pruning, processes essential for cognitive maturation [59]. The resultant neurocognitive deficits, including impairments in processing speed, attention, executive function, and memory, have profound and lasting effects on academic achievement, social functioning, and vocational independence. In contrast, while leukaemia survivors may also receive cranial irradiation (historically for CNS prophylaxis), contemporary protocols have largely replaced radiation with intensified intrathecal chemotherapy, reducing the neurocognitive burden in this population.

Van Gorp et al. (2023) conducted a large national Dutch cohort study of 2154 children with cancer, assessing HRQoL across physical, emotional, social, and school functioning domains using the PedsQL over five years after diagnosis [15]. CNS tumour survivors demonstrated the least favourable HRQoL trajectory, with little improvement over time compared with other diagnostic groups. Among children aged ≥8 years, CNS tumour survivors had consistently lower scores across all four domains, while younger children showed particularly impaired physical and social functioning. Although children with haematological malignancies initially reported poorer HRQoL than some solid-tumour groups, their HRQoL improved more substantially over time. Overall, the findings highlight the particularly persistent HRQoL burden associated with CNS tumours and the importance of long-term, diagnosis-specific supportive care.

The physical domain is particularly affected in CNS tumour survivors, who often contend with motor deficits, visual and hearing impairments, seizures, and endocrine dysfunction, sequelae that are less common or less severe in leukaemia survivors. Psychosocially, CNS tumour survivors face higher rates of social isolation, depression, and anxiety, driven in part by visible physical differences, cognitive limitations, and the challenges of navigating a world that is not designed for individuals with neurocognitive impairments [60]. These consistent differences highlight the need for stratified survivorship care models that allocate resources and interventions according to the specific needs of different diagnostic groups, with CNS tumour survivors requiring the most intensive and multidisciplinary follow-up.

### 5.2. Shared Challenges

Despite these marked differences in HRQoL burden, both leukaemia and CNS tumour survivors share common challenges that underscore the universal impact of childhood cancer on quality of life. Both groups face risks of late effects, neurocognitive difficulties, psychosocial adjustment problems, and compromised educational outcomes, albeit with different profiles and severities. Late effects, including cardiotoxicity from anthracyclines, secondary malignancies, endocrinopathies, and musculoskeletal complications, are a concern for both populations, though the specific patterns differ, leukaemia survivors are at particular risk for metabolic syndrome and cardiac dysfunction, while CNS tumour survivors face greater neurocognitive and endocrine morbidity [61]. Neurocognitive difficulties, while more severe and prevalent in CNS tumour survivors, are also well-documented in leukaemia survivors, particularly those treated with cranial irradiation or high-dose methotrexate, manifesting as subtle deficits in attention, processing speed, and executive function that can impact academic and occupational outcomes. Psychosocial adjustment problems, including anxiety, depression, and post-traumatic stress symptoms, affect both groups, driven by shared experiences of life-threatening illness, intensive treatment, and the challenges of transitioning back to normal life. Both groups report lower HRQoL than healthy populations, though the magnitude of impairment is consistently greater in CNS tumour survivors, reflecting the cumulative burden of neurological, physical, and psychosocial sequelae.

## 6. Determinants of HRQoL

### 6.1. Demographic Factors

Age at diagnosis and sex are consistently identified as important demographic determinants of health-related quality of life (HRQoL) outcomes in childhood cancer survivors. Female childhood cancer survivors have a higher risk for poor HRQoL compared to male survivors, a finding that has been replicated across multiple studies and diagnostic groups. The mechanisms underlying this sex difference are multifactorial and not fully elucidated, but may include biological factors (e.g., hormonal influences on treatment toxicity and recovery), psychological factors (e.g., differences in coping styles, body image concerns, and emotional expression), and social factors (e.g., differential social expectations and support networks) [62]. Females may also be more susceptible to certain treatment-related late effects, such as cardiomyopathy following anthracycline exposure and cognitive impairment following cranial irradiation, which contribute to poorer HRQoL [62]. The impact of age at diagnosis on HRQoL is complex and appears to vary depending on the outcome domain and the specific cancer type. Younger age at diagnosis, particularly under five years, is consistently associated with worse neurocognitive outcomes, especially in children who receive cranial irradiation, reflecting the vulnerability of the developing brain to treatment-related injury. However, the relationship between age at diagnosis and overall HRQoL is more nuanced. Childhood cancer survivors (diagnosed at age 0–14 years) and adolescent/young adult cancer survivors (diagnosed at age 15–39 years) face both shared and distinct challenges. Younger survivors may experience greater neurocognitive and developmental sequelae, with impacts on educational attainment and social development that compound over time. In contrast, adolescent and young adult survivors may face challenges related to the interruption of key developmental milestones, including the transition to independence, formation of intimate relationships, and establishment of career trajectories that affect HRQoL in distinct ways [63]. They may also be more vulnerable to body image concerns, fertility-related distress, and disruptions to educational and vocational trajectories.

### 6.2. Treatment-Related Factors

Treatment intensity, duration, and modality all exert significant influences on HRQoL outcomes in childhood cancer survivors. Cranial irradiation, as noted extensively in the literature, is particularly detrimental, producing dose-dependent neurocognitive impairments that affect processing speed, attention, executive function, and memory, with consequent impacts on academic achievement, social functioning, and overall quality of life. The effects are most pronounced in young children, those receiving higher radiation doses, and those whose radiation fields include critical brain structures such as the hippocampus, temporal lobes, and frontal lobes.

Conversely, treatment protocols that avoid delayed high-dose radiotherapy and marrow ablation chemotherapy show improvement in long-term outcomes, with modern risk-adapted protocols achieving high survival rates while minimising toxicity through reduced radiation doses, targeted therapies, and intensified but less toxic chemotherapy regimens [64]. The shift toward chemotherapy-only protocols for standard-risk ALL, for example, has reduced neurocognitive morbidity while maintaining excellent survival rates. Similarly, the increasing use of proton beam therapy, which delivers less dose to normal tissues, and targeted therapies such as tyrosine kinase inhibitors for Philadelphia chromosome-positive leukaemia, offers the potential to further reduce treatment-related morbidity.

### 6.3. Late Effects

The presence and severity of late effects are among the strongest predictors of HRQoL in childhood cancer survivors, accounting for substantial variance in physical, emotional, and social functioning. Late effects encompass a broad spectrum of physical, neurocognitive, and psychological sequelae that emerge months to decades after treatment completion. Physical late effects include cardiac dysfunction (particularly cardiomyopathy and coronary artery disease following anthracycline exposure and chest radiation), endocrine disorders (growth hormone deficiency, thyroid dysfunction, hypogonadism, and metabolic syndrome), musculoskeletal complications (avascular necrosis, osteoporosis, and scoliosis), pulmonary fibrosis, and secondary malignancies [65]. Each of these physical sequelae has distinct impacts on daily functioning and quality of life; for example, cardiac dysfunction may limit physical activity and contribute to fatigue and reduced participation in sports and recreational activities, while endocrine dysfunction may affect growth, pubertal development, and fertility, with profound implications for body image, self-esteem, and future family planning. Neurocognitive impairments, including deficits in processing speed, attention, executive function, memory, and visuospatial skills, affect academic and vocational achievement, social functioning, and independent living, representing one of the most debilitating classes of late effects [66]. Psychological sequelae, including anxiety, depression, post-traumatic stress symptoms, and social withdrawal, further compound the burden, with some studies suggesting that mental health outcomes may worsen over time as survivors grapple with the cumulative impact of other late effects [67]. The cumulative burden of multiple late effects may be particularly impactful, with survivors experiencing two or more significant late effects reporting substantially lower HRQoL than those with none or one. The concept of “health-related suffering” has been proposed to capture the synergistic impact of multiple comorbidities on quality of life. Importantly, the severity of late effects, rather than their mere presence, appears to be the key determinant of HRQoL, underscoring the need for objective, standardised assessment of late effect severity in clinical practice and research.

### 6.4. Family and Psychosocial Factors

Family functioning and parental adjustment play crucial roles in shaping HRQoL outcomes in childhood cancer survivors, with the family system serving as both a source of resilience and a potential source of distress. Parental distress is robustly correlated with child adjustment and HRQoL, reflecting the reciprocal influences of parent and child wellbeing within the family system. Parents of children with cancer are at risk for impaired HRQoL, with both mothers and fathers reporting significantly impaired functioning across multiple domains, including physical health, emotional wellbeing, social functioning, and role limitations due to emotional problems. The stress of caregiving, financial strain, and concerns about their child’s prognosis and future health contribute to elevated rates of anxiety, depression, and post-traumatic stress symptoms in parents, which in turn affect their ability to provide supportive parenting and to advocate for their child’s needs. Mothers, who often assume the primary caregiver role, may be particularly vulnerable to distress and HRQoL impairment. Low levels of family psychosocial risk are associated with reduced impact of parent psychological distress on child HRQoL, suggesting that resilient families with strong communication, cohesive relationships, and effective problem-solving skills may protect children from the adverse effects of parental distress. Conversely, families with high psychosocial risk—characterised by conflict, poor communication, financial stress, or limited social support—are associated with worse child HRQoL outcomes. Family stress and communication offer potential points of intervention to improve HRQoL, with family-centred interventions such as psychoeducation, cognitive-behavioural therapy, and family therapy showing promise in reducing distress and enhancing family functioning. Sibling adjustment is another important but often overlooked component of family functioning, with siblings of children with cancer experiencing their own distress, including anxiety, jealousy, and feelings of neglect, which can affect family dynamics and overall family HRQoL.

Family stress and communication offer potential points of intervention to improve HRQoL. Family-centred interventions such as psychoeducation, cognitive-behavioural therapy, and family therapy have shown promise in reducing distress and enhancing family functioning. It should be noted, however, that much of the evidence for these interventions derives from broader paediatric psychology and general psychosocial literature, with relatively few large-scale randomised controlled trials conducted specifically in paediatric oncology populations. Further research is needed to establish their efficacy in the childhood cancer context.

### 6.5. Socioeconomic Status

Socioeconomic status (SES) is increasingly recognised as a critical determinant of HRQoL in childhood cancer survivors, reflecting the broader influence of social determinants of health on long-term outcomes. Van Zyl et al. (2024) demonstrated that socioeconomic status is an important determinant of long-term HRQoL among adolescent and young adult childhood cancer survivors in South Africa [68]. Lower socioeconomic status was significantly associated with poorer physical and cognitive functioning, suggesting that socioeconomic disadvantage may persist beyond cancer treatment and influence survivors’ long-term wellbeing. These findings highlight the importance of addressing socioeconomic barriers and ensuring equitable access to survivorship care, rehabilitation, and psychosocial support.

The mechanisms linking SES to HRQoL are multifactorial and include differential access to healthcare, including routine follow-up care, specialised rehabilitation services, and mental health support; differential exposure to health-promoting resources, including healthy nutrition, safe housing, and educational opportunities; and differential stress exposure, including financial strain and neighbourhood violence, which can compound the psychosocial burden of cancer survivorship [69,70].

It is important to acknowledge that the specific mechanisms linking socioeconomic status to HRQoL, particularly regarding health insurance coverage, transportation, and childcare, are context-dependent and may vary considerably across healthcare systems. In countries with universal health coverage, barriers may manifest differently, for example, through longer waiting times, geographic disparities in service availability, or out-of-pocket costs for non-covered services. Nonetheless, the overarching principle that socioeconomic disadvantage is associated with poorer HRQoL outcomes appears consistent across diverse healthcare settings, underscoring the need for equity-oriented approaches to survivorship care globally.

**Figure 1:**
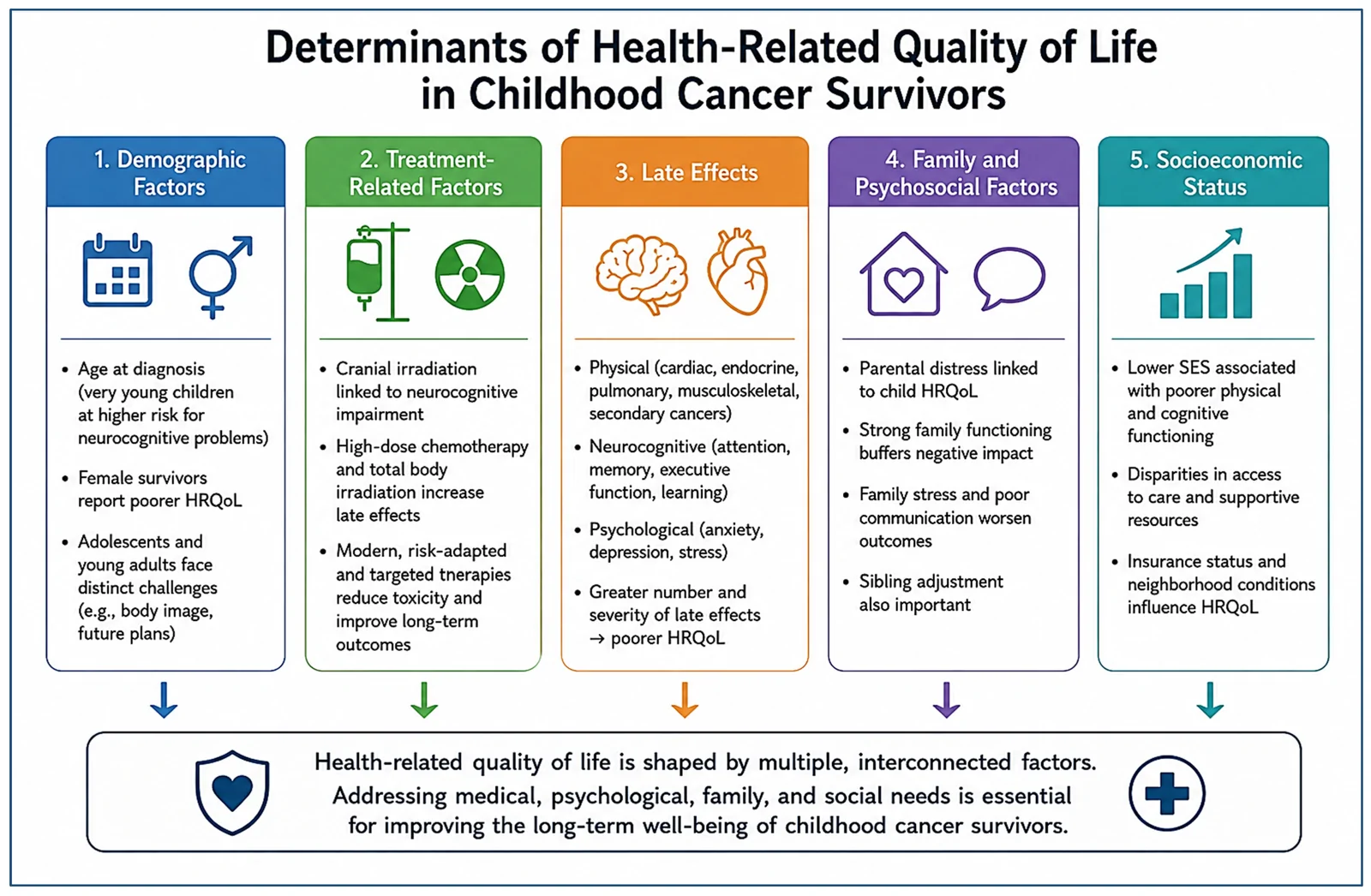
The main determinants of health-related quality of life in childhood cancer survivors. This figure summarises the evidence discussed in Section 6.1, Section 6.2, Section 6.3, Section 6.4 and Section 6.5 of this review. Demographic factors include age at diagnosis and sex; treatment-related factors include cranial irradiation, chemotherapy intensity, and haematopoietic stem cell transplantation; late effects encompass physical, neurocognitive, and psychological sequelae; family and psychosocial factors include parental distress, family functioning, and sibling adjustment; socioeconomic factors include income, insurance status, neighbourhood vulnerability, and healthcare access. See Section 6.1, Section 6.2, Section 6.3, Section 6.4 and Section 6.5 for detailed discussion and supporting references.

Neighbourhood vulnerability, a composite measure of socioeconomic deprivation, crime, and environmental hazards, has also been associated with poor HRQoL among survivors, suggesting that the broader social and physical environment in which survivors live shapes their health and wellbeing (Figure 1).

In the United States, racial and ethnic minority groups are disproportionately affected by lower SES and neighbourhood vulnerability, and studies have documented significant disparities in HRQoL between White and minority survivors. Much of this disparity is accounted for by differences in socioeconomic status; however, Dixon and colleagues found that even after adjusting for current SES, minority survivors continued to report poorer outcomes in some domains [71]. his suggests that while SES is a major contributing factor, other mechanisms, such as cumulative exposure to discrimination, differential treatment experiences, neighbourhood-level factors, or cultural barriers to care, may also play a role. The two findings are complementary rather than contradictory: SES explains a substantial portion of the disparity, but not all of it, pointing to the need for multifaceted interventions that address both material and structural determinants of health.

Complementing these findings, the St. Jude Lifetime Cohort Study showed that greater neighbourhood vulnerability was independently associated with poorer physical and mental HRQoL, even after accounting for individual socioeconomic and clinical factors [72]. Together, these studies emphasize that HRQoL in childhood cancer survivors is influenced not only by medical late effects but also by social, economic, and environmental determinants, supporting the need for equity-focused and socially informed survivorship care.

Healthcare insurance status is another important factor, with uninsured and underinsured survivors facing barriers to accessing survivorship care, including late-effects screening and treatment, which may contribute to poorer HRQoL (Figure 1). These findings underscore the importance of addressing health disparities in survivorship care through targeted interventions that address the social determinants of health, including expanding access to healthcare through insurance coverage, providing transportation and childcare support to facilitate clinic attendance, and offering culturally sensitive psychosocial support. Policy-level interventions that address economic inequality, housing insecurity, and educational disparities are also essential for reducing socioeconomic disparities in HRQoL (Figure 1).

### 6.6. Interventions to Improve HRQoL

Several intervention approaches have been investigated to improve HRQoL in childhood cancer survivors, though the evidence base remains preliminary and predominantly observational.

Physical activity interventions have shown promise in mitigating treatment-related fatigue, improving cardiovascular fitness, and enhancing psychosocial wellbeing. Supervised exercise programmes during and after treatment are associated with better physical HRQoL, although adherence remains challenging, particularly during intensive treatment phases [73].

Psychosocial and psychological interventions, including cognitive-behavioural therapy, psychoeducation, and family-based programmes, have demonstrated benefits for emotional and social HRQoL domains. Family-centred interventions targeting communication, problem-solving, and parental distress are associated with improved child adjustment; however, most studies are small and lack long-term follow-up.

Cognitive rehabilitation programmes, including computerised cognitive training, memory strategies, and attention-processing interventions, have shown moderate effects on neurocognitive outcomes in brain tumour survivors. Evidence for translation to real-world functional improvements and overall HRQoL remains limited.

Digital health technologies, such as mobile applications for symptom monitoring, telehealth platforms for survivorship follow-up, and online psychosocial support, represent emerging approaches that may increase access to care and enable real-time HRQoL monitoring. Early evidence suggests acceptability and feasibility, though efficacy data are still emerging.

Importantly, the majority of intervention studies in this population are single-centre, underpowered, and lack control groups. There is a pressing need for larger, multicentre randomised controlled trials with standardised HRQoL outcomes to establish a robust evidence base.

## 7. Future Directions

Looking ahead, advancing the field of health-related quality of life in paediatric oncology demands a multifaceted strategy that spans methodological standardisation, clinical translation, targeted intervention, robust evidence generation, and a fundamental reorientation of survivorship goals. A cornerstone of this future vision is the establishment of core outcome sets for HRQoL assessment, with the extensively validated and widely adopted PedsQL emerging as a prime candidate to serve as a unifying benchmark. Such standardisation is not merely an academic exercise; it is the essential prerequisite for embedding HRQoL screening into routine clinical workflows and longitudinal survivorship care. By implementing regular, systematic assessments, modelled on the tiered approach already proposed for paediatric brain tumour survivors, clinicians can proactively identify individuals at heightened risk and trigger timely, targeted supportive interventions before problems become entrenched. The data gleaned from these assessments must then inform a paradigm shift from generic supportive care towards precisely personalised interventions. Rather than applying a uniform approach, future strategies should be dynamically tailored to each child’s unique profile, integrating their specific diagnosis, treatment history, cumulative late-effects burden, and pre-existing psychosocial vulnerabilities to maximise clinical benefit.

To validate and refine these personalised approaches, however, the field must urgently invest in large-scale, prospective, multisite collaborative networks. Only through such longitudinal designs can we confidently disentangle the complex interplay of disease and treatment modalities, identify genuinely modifiable risk factors across different populations, and evaluate the efficacy of novel interventions under real-world conditions. Crucially, this evidence base must be built and applied through an unwavering lens of health equity. Persistent disparities linked to socioeconomic status, racial and ethnic background, and geographic location must be actively dismantled to ensure that high-quality, comprehensive survivorship care is universally accessible, regardless of a family’s circumstances.

Ultimately, however, these collective efforts must converge on a transformative overarching goal: shifting the focus beyond biological survival toward the assurance of meaningful life participation. HRQoL metrics and interventions must therefore be recalibrated to capture and foster not just physical health, but robust educational attainment, vocational fulfilment, stable interpersonal relationships, and sustained emotional well-being. In doing so, paediatric oncology can fulfil its ultimate objective, not merely extending survival but optimising the quality of that survival for every child and family touched by cancer.

## 8. Conclusions

The remarkable improvements in survival for children with acute leukaemias and CNS tumours have been accompanied by growing recognition of the importance of health-related quality of life as a critical outcome. The evidence reviewed here demonstrates that while children with ALL generally experience HRQoL comparable to or modestly below population norms, children with CNS tumours face substantially greater and more persistent impairments. These differences reflect the direct impact of CNS pathology and treatment on the developing brain, the neurocognitive and psychosocial sequelae that ensue, and the cumulative burden of multiple late effects. Key determinants of HRQoL include treatment-related factors (particularly cranial irradiation), late effect burden, family functioning, parental distress, and socioeconomic status. Promising interventions include physical activity programmes, psychosocial support, cognitive rehabilitation, and digital health technologies. However, significant methodological challenges, including measurement heterogeneity, limited longitudinal data, and underrepresentation of key populations, must be addressed.

Future research should prioritise standardised HRQoL assessment, integration into routine clinical care, personalised interventions, longitudinal multisite studies, and health equity.

## Figures and Tables

**Table 1:** Measuring HRQoL in Paediatric Oncology: Framework, Instruments, and Challenges.

Domain	Key Points	Challenges	Recommendations	Ref
Conceptual Framework	Multidimensional construct (physical, psychological, social, functional domains) plus developmental considerations; domain salience shifts with age (younger: physical/pain; adolescents: social/school/autonomy)	Disease/treatment impact mediated by developmental tasks; dynamic interplay between age and HRQoL	Adopt lifespan developmental perspective; consider both chronological and developmental age in assessment	[16]
Assessment Instruments	PedsQL 4.0 & Cancer Module: Most widely used; α = 0.82–0.96; validated cross-culturally	≥12 distinct tools identified across studies; heterogeneity limits comparability and meta-analytic synthesis	Prioritise PedsQL for cross-cultural comparability; select instruments based on study objectives; work toward harmonised consensus	[10]
	CHQ: Broad physical/psychosocial coverage			[17]
	HUI: Preference-based utility scores for cost-utility analyses			[18]
	POQOLS: Cancer-specific perspective			[7]
Self-Report vs. Proxy-Report	Children ≥5 years can self-report reliably; parents report more impairment (especially psychosocial domains); perspectives are complementary, not competing	Parent–child agreement is moderate; discordance may reflect parental distress, caregiver burden, or protective projection	Obtain both informants whenever feasible; use self-report for subjective experience, proxy for external longitudinal overview; address discordance as potential marker of unmet needs	[12]
Methodological Challenges	Instrument heterogeneity: Limits comparability and meta-analyses	Hampers interpretation of change, intervention effectiveness, and generalisability; may perpetuate disparities	Establish population-specific MIDs; prioritise longitudinal designs; develop culturally/linguistically validated instruments; include vulnerable subgroups	[19]
	Missing MIDs: No consensus on clinically meaningful differences			[20]
	Cross-sectional predominance: Longitudinal studies scarce			[21]
	Under-representation: Infants, adolescents, LMIC patients often excluded			[22]
Core Recommendations	Routine HRQoL assessment as core patient-reported outcome; longitudinal tracking from diagnosis through survivorship; dual informant (child + parent); harmonised instrument selection; cultural validation for cross-cultural research	Persistent challenges require consensus-building and methodological standardisation	Integrate screening into clinical practice; conduct multi-centre longitudinal studies; adopt core outcome sets; validate tools across diverse populations	[7]

**Table 2:** HRQoL in Paediatric ALL and CNS Tumours: Key Findings.

Disease Group	Phase	Key HRQoL Findings	Key Determinants/Associations	Implications	Ref
ALL	During Treatment	Substantial impairment across all domains; physical domain most affected (fatigue, pain, nausea, weight gain); lowest scores during induction; some recovery in maintenance	Treatment intensity; corticosteroids; vincristine neuropathy; hospitalisations; social isolation	Routine HRQoL monitoring; integrated psychosocial support; symptom management protocols	[54]
	Early Post-Treatment (≤5 yrs)	Significant improvement vs. active treatment, but remains below population norms; emergence of late effects; post-treatment fatigue common	Late effects (neurocognitive, endocrine, cardiac); cranial irradiation; female sex; older age; family dysfunction; parental distress	Targeted interventions: cognitive rehab, psychological support, transition care	[28]
	Long-Term Survivorship	Variable: 13/31 studies found worse HRQOL, 8 no difference, 3 better; ALL survivors generally better than CNS tumour/sarcoma survivors; “response shift” phenomenon	Worse late effects (strongest predictor); psychological factors (resilience, depression); socio-demographic factors; NOT consistently associated with treatment received	Longitudinal assessment; comprehensive risk factor evaluation; early intervention strategies	[55]
AML	All Phases	Limited evidence; early HRQoL impairment predicts long-term prognosis; outpatient neutropenia management not associated with worse HRQoL	Lower household income (selection bias concerns); treatment intensity (HSCT, high-dose cytarabine)	Larger multicentre prospective studies needed; HRQoL as prognostic tool	[35]
CNS Tumours	All Phases	Worse HRQoL than healthy comparisons (MD −0.54, 95% CI −0.72 to −0.35) and non-CNS cancer survivors (MD −0.56, 95% CI −0.73 to −0.38); deficits across all domains; lifelong disability common	Tumour location (infratentorial: cerebellar mutism; supratentorial: focal cognitive deficits); cranial irradiation (dose to hippocampus/pituitary); younger age at treatment; proton vs. photon therapy	Radiation-sparing strategies (proton therapy); cognitive rehabilitation; lifelong multidisciplinary follow-up	[43]
	Neurocognitive/Psychosocial	Deficits in processing speed, attention, executive function, memory, visuospatial skills; “growing into deficit” phenomenon; low self-esteem, depression, suicidal ideation; poor educational/vocational outcomes	Cranial irradiation (dose-dependent); treatment modality (proton better than photon but not risk-free); early-stage radiotherapy	Cognitive remediation therapy; pharmacological support (memantine, donepezil); educational accommodations	[56]
	Tumour Location	Infratentorial: cerebellar mutism (speech impairment, ataxia, emotional lability); Supratentorial: site-specific deficits (frontal: executive; temporal: memory/language; parietal: visuospatial); medulloblastoma: severe phenotype	Cerebellar damage; craniospinal irradiation; surgical resection extent	Targeted rehabilitation strategies; family counselling on expected outcomes	[57]
	Cranial Irradiation	Dose-dependent deficits in HRQoL; hippocampus (≥10 Gy) and pituitary gland most sensitive; radiation-induced vascular injury	Radiation dose to brain substructures; younger age	Proton therapy; IMRT; neuroprotective agents (memantine, donepezil); careful radiation planning	[52]

## Data Availability

No new data were created or analyzed in this study. Data sharing is not applicable to this article.

## References

[1] Roganovic J. (2025). Late effects of the treatment of childhood cancer. World J. Clin. Cases.

[2] Rocka A., Lesniak M., Majka P., Lipniarska J., Suchcicka M., Lejman M., Wozniak M., Zawitkowska J. (2025). Cerebrovascular complications in pediatric patients with acute lymphoblastic leukemia. Sci. Rep..

[3] Santero M., Sequeira R.O., Paixao M.C., Martinez M.M., Roig P.M., Chantada G., La Madrid A.M., Ilbawi A. (2025). Childhood cancer survival in low- and middle-income countries and the Global South: Emerging evidence and critical gaps from a scoping review of observational studies. EJC Paediatr. Oncol..

[4] Damodharan S., Puccetti D. (2023). Pediatric Central Nervous System Tumor Overview and Emerging Treatment Considerations. Brain Sci..

[5] Davies M.R., Greenberg Z., van Vuurden D.G., Cross C.B., Zannettino A.C., Bardy C., Wardill H.R. (2023). More than a small adult brain: Lessons from chemotherapy-induced cognitive impairment for modelling paediatric brain disorders. Brain Behav. Immun..

[6] Borowska M.W., Sikora-Koperska A. (2025). The importance of health-related quality of life (HRQoL) assessment in oncology care for adolescents and young adults (AYA). Nowotw. J. Oncol..

[7] Horan M.R., Sim J.-A., Krull K.R., Baker J.N., Huang I.-C. (2022). A Review of Patient-Reported Outcome Measures in Childhood Cancer. Children.

[8] Wang J., Jin W., Shi L., Geng Y., Zhu X., Hu W. (2022). Health-Related Quality of Life in Children: The Roles of Age, Gender and Interpersonal Trust. Int. J. Environ. Res. Public Health.

[9] Kaur M., Kaur S. (2023). Concept of quality of life in health care research: A review. Int. J. Community Med. Public Health.

[10] Irestorm E., Bakker A., Tissing W.J.E., Maurice-Stam H., Dors N., Mavinkurve-Groothuis A., Plasschaert S.L.A., van Bindsbergen K.L.A., Grootenhuis M.A., van Litsenburg R.R.L. (2025). Benchmarking the pediatric quality of life (PedsQL) cancer module in a large Dutch national cohort of childhood cancer patients. BMC Cancer.

[11] Khanna D., Khadka J., Mpundu-Kaambwa C., Lay K., Russo R., Ratcliffe J., Devlin N., Norman R., Viney R., Dalziel K. (2022). Are We Agreed? Self- Versus Proxy-Reporting of Paediatric Health-Related Quality of Life (HRQoL) Using Generic Preference-Based Measures: A Systematic Review and Meta-Analysis. PharmacoEconomics.

[12] Mack J.W., McFatrich M., Withycombe J.S., Maurer S.H., Jacobs S.S., Lin L., Lucas N.R., Baker J.N., Mann C.M., Sung L. (2020). Agreement Between Child Self-report and Caregiver-Proxy Report for Symptoms and Functioning of Children Undergoing Cancer Treatment. JAMA Pediatr..

[13] Rothmund M., Meryk A., Rumpold G., Crazzolara R., Sodergren S., Darlington A.-S., Riedl D., the EORTC Quality of Life Group (2023). A critical evaluation of the content validity of patient-reported outcome measures assessing health-related quality of life in children with cancer: A systematic review. J. Patient-Rep. Outcomes.

[14] Bamber R., Stavroulakis T., McDermott C., Carlton J. (2025). Health-related quality of life of informal carers in ALS: A systematic review of person reported outcome measures. Qual. Life Res..

[15] van Gorp M., Irestorm E., Twisk J.W., Dors N., Mavinkurve-Groothuis A., Meeteren A.Y., De Bont J., van den Bergh E.M., van der Meer W.V., Beek L.R. (2023). The course of health-related quality of life after the diagnosis of childhood cancer: A national cohort study. BMC Cancer.

[16] Christou A.I., Kalfadeli G., Bacopoulou F. (2026). Developmental Foundations of Psychosocial Interventions in Pediatric Oncology: A Lifespan Framework for Resilience. Children.

[17] Sansom-Daly U.M., McLoone J.K., Fardell J.E., Evans H.E., McGill B.C., Robertson E.G., Signorelli C., Ellis S., Ha L., Hetherington K. (2025). Bridging the Gap: Embedding Psychosocial Oncology Research into Comprehensive Cancer Care for Children and Young People. Cancers.

[18] Viani K., Furlong W., Filho V.O., Murra M.d.S., Nabarrete J.M., Ladas E., Barr R.D. (2024). Comprehensive health status and health-related quality of life of children at diagnosis of high-risk neuroblastoma: A multicentric pilot study. Hematol. Transfus. Cell Ther..

[19] Heller N., Melnikov S. (2024). Factors affecting caregiver burden among parents of children with cancer: A path analysis. J. Clin. Nurs..

[20] Zou X., Srivastava T., Hetherington K., Yu A., Marshall G.M., Mateos M.K. (2025). Factors that influence the uptake of precision-guided treatment recommendations in paediatric cancer: A systematic review. Br. J. Cancer.

[21] Mikkelsen M.K., Pham L.T.D., Sotelo C., Kelley M., Edwards M., Lion R.R., Ravi H., Chantada G., Lucas J.T., Robinson G.W. (2026). The Global Clinical Trial Landscape for Children and Adolescents with Cancer. JAMA Netw. Open.

[22] Klootwijk L., Apadet L., Njuguna F., Nzamu I., Chagaluka G., Ardianto B., Sari N.M., Majaliwa E.L., Zejnullahu A.B., Huibers M. (2024). Delays in access to paediatric oncology care: Perspectives from eight partner hospitals. S. Afr. J. Child Health.

[23] Thastrup M., Duguid A., Mirian C., Schmiegelow K., Halsey C. (2022). Central nervous system involvement in childhood acute lymphoblastic leukemia: Challenges and solutions. Leukemia.

[24] Ospina P.A., Al Onazi M.M., McNeely M.L. (2025). Physical therapy for deficits associated with chemotherapy induced peripheral neuropathy in children with cancer: A systematic review. Pediatr. Med..

[25] Lee N.W., Lee K.H. (2024). Methotrexate-Induced Stroke-Like Leukoencephalopathy. Ann. Child Neurol..

[26] Amir Y., Mahamid S., Li J., Barzilai-Birenboim S., Hayek S. (2026). Cancer therapy and reproductive health in childhood cancer survivors: A systematic review of long-term effects. F S Rev..

[27] Ashaye A., Shi L., Aldoss I., Montesinos P., Vachhani P., Rocha V., Papayannidis C., Leonard J.T., Baer M.R., Ribera J.-M. (2025). Impact of clinical response and treatment tolerability on HRQoL in newly diagnosed Philadelphia chromosome-positive acute lymphoblastic leukemia patients treated with ponatinib or imatinib. Ann. Hematol..

[28] Kızmazoğlu D., Sarı S., Evim M.S., Kantarcıoğlu A.Ç., Tüfekçi Ö., Yenigürbüz F.D., Baytan B., Yılmaz S., Güneş A.M., Ören H. (2019). Assessment of Health-Related Quality of Life in Pediatric Acute Lymphoblastic Leukemia Survivors: Perceptions of Children, Siblings, and Parents. Turk. J. Hematol..

[29] Dirven L., Luerding R., Beier D., Bumes E., Reinert C., Seidel C., Bonsanto M.M., Bremer M., Rieken S., Combs S.E. (2020). Neurocognitive functioning and health-related quality of life in adult medulloblastoma patients: Long-term outcomes of the NOA-07 study. J. Neuro-Oncol..

[30] Li M., Yang L., Liu S., Chen L., Ma C.C. (2023). Endocrine Late Effects of Radiation Therapy in Pediatric and Young Adult Patients with Brain Tumors. Mathews J. Cancer Sci..

[31] Andreu Y., Picazo C., Soto-Rubio A., Gil-Julia B. (2025). Health-related quality of life in cancer survivors aged 65 and older: Impact of diagnosis and treatment and perceived decline associated with aging. Support. Care Cancer.

[32] Vetsch J., Wakefield C.E., Robertson E.G., Trahair T.N., Mateos M.K., Grootenhuis M., Marshall G.M., Cohn R.J., Fardell J.E. (2018). Health-related quality of life of survivors of childhood acute lymphoblastic leukemia: A systematic review. Qual. Life Res..

[33] Chantziara S., Musoro J., Rowsell A.C., Sleurs C., Coens C., Pe M., Suciu S., Kicinski M., Missotten P., Vandecruys E. (2022). Quality of life of long-term childhood acute lymphoblastic leukemia survivors: Comparison with healthy controls. Psycho-Oncology.

[34] Jia F., Li Y., Hao T., Ma G. (2025). Epidemiology of acute myeloid leukemia in children and adolescents (1990–2021): A global burden of disease study. Sci. Nat..

[35] Wu T., Fu W., Xue Y., Zhu L., Ma X., Wei Y., Li H., Wang Y., Kang M., Fang Y. (2024). Health-related quality of life in children with childhood acute myeloid leukemia in China: A five-year prospective study. Heliyon.

[36] Getz K.D., Szymczak J.E., Li Y., Madding R., Huang Y.-S.V., Aftandilian C., Arnold S.D., Bona K.O., Caywood E., Collier A.B. (2021). Medical Outcomes, Quality of Life, and Family Perceptions for Outpatient vs Inpatient Neutropenia Management After Chemotherapy for Pediatric Acute Myeloid Leukemia. JAMA Netw. Open.

[37] Pawińska-Wąsikowska K., Czogała M., Bukowska-Strakova K., Surman M., Rygielska M., Książek T., Sadowska B., Pac A., Skalska-Sadowska J., Samborska M. (2024). Treatment Outcomes of Adolescents Compared to Younger Pediatric Patients with Acute Myeloid Leukemia: Do They Need a Special Approach?. Cancers.

[38] Tilg A., Kuhle S., Meryk A., Hetzer B., Kropshofer G., Salvador C., Weiss J.G., Lehmann J., Riedl D., Rumpold G. (2025). Cancer-related fatigue in children during treatment: A 5-year cohort study of daily patient-reported outcomes with clinical implications. eClinicalMedicine.

[39] Su Z., Lu J., Shi Y., Li T., Qi B., Guo Z. (2025). Global, regional, and national childhood brain and central nervous system cancer burden: An analysis based on the Global Burden of Disease Study. Trop. Med. Health.

[40] Samman R.R., Timraz J.H., Al-Nakhli A.M., Haidar S., Muhammad Q., Thalib H.I., Mousa A.H., Kharoub M.S. (2024). The Impact of Brain Tumors on Emotional and Behavioral Functioning. Cureus.

[41] Piec K., Blok M., Adamczak-Sobczak M., ZaręBska I., Harat M. (2026). Cognitive Impairment in Patients with Glioma: Mechanisms, Assessment, and Emerging Therapeutic Strategies. Cancers.

[42] Baqai M.W.S., Tariq R., Shah Z., Bajwa M.H., Shamim M.S. (2023). Quality of life in survivors of pediatric medulloblastoma: A systematic review and meta-analysis. Child’s Nerv. Syst..

[43] Rüther M., Hagan A.J., Verity S.J. (2023). The role of CNS tumor location in health-related quality of life outcomes: A systematic review of supratentorial vs infratentorial tumors in childhood survivorship. Appl. Neuropsychol. Child.

[44] Rothmund M., Sodergren S., Rohde G., de Rojas T., Paratico G., Albini G., Mur J., Darlington A.-S., Majorana A., Riedl D. (2022). Updating our understanding of health-related quality of life issues in children with cancer: A systematic review of patient-reported outcome measures and qualitative studies. Qual. Life Res..

[45] Christou A.I., Kalfadeli G., Tsermentseli S., Bacopoulou F. (2025). Neurocognitive and Emotional Outcomes in Childhood Cancer: A Developmental Perspective. Curr. Oncol..

[46] Mavrea K., Katsibardi K., Roka K., Pons R., Efthymiou V., Antoniou A.-S., Christou A.I., Kanaka-Gantenbein C., Chrousos G.P., Kattamis A. (2026). Cognitive and Psychosocial Burden of Childhood Cancer Survivors in Greece: A Case–Control Study. Med. Sci..

[47] Lassaletta Á., Morales J.S., Valenzuela P.L., Esteso B., Kahalley L.S., Mabbott D.J., Unnikrishnan S., Panizo E., Calvo F. (2023). Neurocognitive outcomes in pediatric brain tumors after treatment with proton versus photon radiation: A systematic review and meta-analysis. World J. Pediatr..

[48] Mash L.E., Kahalley L.S., Raghubar K.P., Goodrich-Hunsaker N.J., Abildskov T.J., De Leon L.A., MacLeod M., Stancel H., Parsons K., Biekman B. (2023). Cognitive Sparing in Proton versus Photon Radiotherapy for Pediatric Brain Tumor Is Associated with White Matter Integrity: An Exploratory Study. Cancers.

[49] Heinzel A., Mottaghy F.M., Filss C., Stoffels G., Lohmann P., Friedrich M., Shah N.J., Caspers S., Lucas C.W., Ruge M.I. (2023). The impact of brain lesions on health-related quality of life in patients with WHO CNS grade 3 or 4 glioma: A lesion-function and resting-state fMRI analysis. J. Neuro-Oncol..

[50] Morosan G.-C., Moroșan A.-C., Ionescu C., Sava A. (2024). Neuropsychiatric symptoms as early indicators of brain tumors. Arch. Clin. Cases.

[51] Castell A.F.P., Rodríguez J.d.l.S.R., Mata E.M., Facio E.L., López F.K.S. (2026). Children with Medulloblastoma: Surgical Outcomes and Survival Challenges. Cureus.

[52] Doig M., Lee J., Kwok Y., MacEwan I., Wolden S., Allison K., Dennehy S., Bajaj B., Short M., Gorayski P. (2025). Impact of substructure radiation dose on health-related quality of life in children with brain tumors: A Pediatric Proton/Photon Consortium Registry (PPCR) study. J. Neuro-Oncol..

[53] McLaren D.S., Devi A., Kyriakakis N., Kwok-Williams M., Murray R.D. (2023). The impact of radiotherapy on the hypothalamo-pituitary axis: Old vs new radiotherapy techniques. Endocr. Connect..

[54] Tager J.B., Palou-Torres A., Bingen K.M., Hoag J.A., Burke M.J., Zhang J., Yan K., Karst J.S. (2024). Health-related quality of life among pediatric patients with acute lymphoblastic leukemia: An exploratory cross-sectional study. Pediatr. Blood Cancer.

[55] Muffly L., Maguire F.B., Li Q., Kennedy V., Keegan T.H. (2020). Late Effects in Survivors of Adolescent and Young Adult Acute Lymphoblastic Leukemia. JNCI Cancer Spectr..

[56] Sciancalepore F., Fabozzi F., Albino G., Del Baldo G., Di Ruscio V., Laus B., Menegatti D., Premuselli R., Secco D.E., Tozzi A.E. (2023). Frequency and characterization of cognitive impairments in patients diagnosed with paediatric central nervous system tumours: A systematic review. Front. Oncol..

[57] Salman A., Fahim M.A.A., Sohail A., Bashir H.H., Haizel-Cobbina J., Dewan M.C., Shamim M.S. (2026). Pre-operative and intra-operative risk factors of post-operative cerebellar mutism syndrome in pediatric patients undergoing posterior fossa tumor surgery: A systematic review. Child’s Nerv. Syst..

[58] Pancaldi A., Pugliese M., Migliozzi C., Blom J., Cellini M., Iughetti L. (2023). Neuropsychological Outcomes of Children Treated for Brain Tumors. Children.

[59] Lee S., Lucas S., Proudman D., Nellesen D., Paulose J., Sheehan V.A. (2022). Burden of central nervous system complications in sickle cell disease: A systematic review and meta-analysis. Pediatr. Blood Cancer.

[60] Kumar R., Narra L.R., Sherwani Z., Parikh R.R. (2025). Neurological sequalae in pediatric patients with CNS tumors after radiation treatment: A comprehensive review. Semin. Pediatr. Neurol..

[61] Winzig J., Inhestern L., Paul V., Nasse M.L., Krauth K.A., Kandels D., Rutkowski S., Escherich G., Bergelt C. (2023). Parent-reported health-related quality of life in pediatric childhood cancer survivors and factors associated with poor health-related quality of life in aftercare. Qual. Life Res..

[62] Han X., Robinson L.A., Jensen R.E., Smith T.G., Yabroff K.R. (2021). Factors Associated with Health-Related Quality of Life Among Cancer Survivors in the United States. JNCI Cancer Spectr..

[63] Rhodes M., Signorelli C., Donoghoe M.W., McLoone J.K., Wakefield C.E., Anazodo A., Sansom-Daly U.M., Darlington A.-S., Sodergren S.C., Cohn R.J. (2026). Factors associated with health-related quality of life in survivors of childhood-onset and adult-onset cancer compared to controls. Support. Care Cancer.

[64] Pearlman R., Hanna R., Burmeister J., Abrams J., Dominello M. (2021). Adverse Effects of Total Body Irradiation: A Two-Decade, Single Institution Analysis. Adv. Radiat. Oncol..

[65] Signorelli C., Wakefield C.E., McLoone J.K., Johnston K.A., Mertens A.C., Osborn M., Cohn R.J. (2023). Childhood Cancer Survivors’ Reported Late Effects, Motivations for Seeking Survivorship Care, and Patterns of Attendance. Oncologist.

[66] Castillo R.L., Figueroa E.G., González-Candia A., del Campo A., Paris C., Verdugo F., Lang M., Cruz-Montecinos C., Quezada M., Pérez R.A. (2025). Effects of Exercise on Cardiovascular and Metabolic Responses in Adults and Childhood Cancer Survivors: The Role of NETosis and Low-Grade Inflammation as a Novel Therapeutic Target—A Narrative Review. Int. J. Mol. Sci..

[67] Maas A., Maurice-Stam H., Feijen E.A.M., Teepen J.C., Delden A.M.v.d.A., Streefkerk N., Broeder E.v.D., Tissing W.J.E., Loonen J.J., van der Pal H.J.H. (2024). Risk and Protective Factors of Psychosocial Functioning in Survivors of Childhood Cancer: Results of the DCCSS-LATER Study. Psycho-Oncology.

[68] Van Zyl A., Kruger M., Ndlovu S., Rogers P.C. (2024). Health-Related Quality of Life of Adolescent and Young Adult-Aged Childhood Cancer Survivors in a South African Cohort: A Pilot Study Using the Minneapolis-Manchester Quality of Life Instrument. J. Adolesc. Young Adult Oncol..

[69] Mai Q., Xu S., Hu J., Sun X., Chen G., Ma Z., Song Y., Wang C. (2023). The association between socioeconomic status and health-related quality of life among young and middle-aged maintenance hemodialysis patients: Multiple mediation modeling. Front. Psychiatry.

[70] Hamasaki H. (2025). The Relationship Between Socioeconomic Status and Health Behaviors in Older Adults: A Narrative Review. Healthcare.

[71] Dixon S.B., Li N., Yasui Y., Bhatia S., Casillas J.N., Gibson T.M., Ness K.K., Porter J.S., Howell R.M., Leisenring W.M. (2019). Racial and ethnic disparities in neurocognitive, emotional, and quality-of-life outcomes in survivors of childhood cancer: A report from the Childhood Cancer Survivor Study. Cancer.

[72] Choi J., Horan M.R., Brinkman T.M., Srivastava D.K., Ness K.K., Armstrong G.T., Hudson M.M., Huang I.-C. (2024). Neighborhood vulnerability and associations with poor health-related quality of life among adult survivors of childhood cancer. JNCI Cancer Spectr..

[73] Ortiz-Comino L., Abril-Mera T.M., Fernández-Gualda M.Á., Lozano-Lozano M., Herbawi F., Fernández-Lao C. (2026). Benefits of Physiotherapy Interventions in Survivors of Childhood Cancer: A Systematic Review with Meta-Analysis. Cancers.

